# Anti-Cancer Natural Products and Their Bioactive Compounds Inducing ER Stress-Mediated Apoptosis: A Review

**DOI:** 10.3390/nu10081021

**Published:** 2018-08-04

**Authors:** Changmin Kim, Bonglee Kim

**Affiliations:** Department of Pathology, College of Korean Medicine, Graduate School, Kyung Hee University, 1 Hoegi-dong, Dongdaemun-gu, Seoul 130-701, Korea; ckdals4302@khu.ac.kr

**Keywords:** natural products, bioactive compounds, cancer, endoplasmic-reticulum stress, unfolded protein response, apoptosis

## Abstract

Cancer is the second biggest cause of death worldwide. Despite a number of studies being conducted, the effective mechanism for treating cancer has not yet been fully understood. The tumor-microenvironment such as hypoxia, low nutrients could disturb function of endoplasmic reticulum (ER) to maintain cellular homeostasis, ultimately leading to the accumulation of unfolded proteins in ER, so-called ER stress. The ER stress has a close relation with cancer. ER stress initiates unfolded protein response (UPR) to re-establish ER homeostasis as an adaptive pathway in cancer. However, persistent ER stress triggers the apoptotic pathway. Therefore, blocking the adaptive pathway of ER stress or facilitating the apoptotic pathway could be an anti-cancer strategy. Recently, natural products and their derivatives have been reported to have anti-cancer effects via ER stress. Here, we address mechanisms of ER stress-mediated apoptosis and highlight strategies for cancer therapy by utilizing ER stress. Furthermore, we summarize anti-cancer activity of the natural products via ER stress in six major types of cancers globally (lung, breast, colorectal, gastric, prostate and liver cancer). This review deepens the understanding of ER stress mechanisms in major cancers as well as the suppressive impact of natural products against cancers via ER stress.

## 1. Introduction

Cancer is a group of diseases that undergo unregulated cell growth and proliferation without stopping [1]. Although overall survival term is increased slightly due to early detection, cancer-related mortality is the second biggest cause of death worldwide [2]. The International Agency for Research on Cancer (IARC) reported that 14.1 million new cancer cases and 8.2 million deaths took place worldwide in 2012, and 21.7 million cancer incidences and 13 million deaths were predicted in 2030. Among several types of cancers, lung, breast, colorectal, gastric, prostate, and liver cancer were selected as the major cancers in human being. These cancers represent 55% of the global cancer incidence burden in 2012 [3].

The mechanisms of cancer occurrence and progression are not fully understood yet. The proliferation of cancer originated from its ability to avoid programmed cell death, so-called apoptosis [4]. That is why induction of apoptosis in cancer has been identified as a target for treatment of cancer [5,6]. Up to now, induction of apoptosis is conducted by two main apoptotic pathways: intrinsic and extrinsic pathway [7]. Intrinsic pathway is mitochondria-mediated apoptosis which is mediated by cytochrome C release and activation of caspase-9, stimulating effector caspases, caspase-3. Extrinsic pathway is death receptor (DR)-mediated apoptosis which activates the FAS-associated death domain (FADD) and forms the death-inducing signaling complex (DISC), which processes downstream caspases including caspase-8,-7,-6, and -3 [8,9]. However, studies have identified that the accumulation of unfolded proteins in endoplasmic reticulum (ER) and its cellular stress response is involved in apoptosis, indicating influence of ER on cell fate as a third subcellular compartment [10].

### Overview of ER Stress; A Double-Edged Sword-Cell Survival or Death?

ER is an organelle in the eukaryotic cell that is responsible for protein synthesis and calcium (Ca^2+^) signaling [11]. It also provides a suitable environment for lipid, steroid, and cholesterol biosynthesis. Moreover, the main roles of ER include maintenance of homeostasis in intracellular Ca^2+^ storage and the folding of protein destined to be secreted on the plasma membrane [12]. Proteins are translocated into ER lumen and undergo post translational modification for fidelity of synthesis, folding and correcting function. After passing ER, proteins are properly folded by a network of chaperones [13].

However, extra-cellular environmental challenges such as reactive oxygen species (ROS), hypoxia, and nutrients deprivation could induce disturbance in cellular redox regulation of ER, leading to an imbalance in homeostasis. Thus, the physiological function of ER and the ER protein-folding environment are impaired, ultimately resulting in the accumulation of unfolded protein in the lumen of the ER-namely, ER stress [10]. Prior studies have demonstrated that both ER stress and the activation of unfolded protein response (UPR) are associated with pathologic processes, including neurodegenerative diseases, cardiovascular disease, and cancer [14]. Because of the rapid expansion of malignant neoplasm, cancer cells are exposed to low nutrients, poor vascularization, and hypoxia so that ER stress-related proteins including glucose-regulated protein 78 (GRP78)/binding protein (BiP), glucose-regulated protein 94 (GRP 94), ER associated degradation (ERAD)*,* protein disulphide isomerase (PDI), activating transcription factor 6 (ATF6), inositol-requiring protein 1 (IRE1), α-x-box binding protein 1 (XBP1), protein kinase RNA-like ER kinase (PERK) and eukaryotic initiation factor 2 (eIf2α) are overexpressed in many types of cancer cells [15,16,17]. In response to ER stress on early phase, the cell initiates UPR to modulate proper protein folding and degradation of unfolded protein as an adaptive pathway for survival. Under ER stress, GRP78/BiP, which originally binds to the luminal domain of three ER transducer sensors: IRE1, PERK, and ATF6, dissociates from these three ER transducer sensors. The three ER-localized transmembrane signal-transducers of UPR; ATF6, IRE1 and PERK detect accumulation of unfolded proteins and initiate to restore and maintain the ER homeostasis [18]. Moreover, ERAD is increased to attenuate unfolded protein accumulation, enhancing protein folding capacity in ER and ER chaperones (GRP 94, GRP78/BiP, calnexin), which are elevated to stabilize protein folding. However, if ER stress is prolonged to the extent that UPR is unable to cope with unfolded proteins, UPR is promoted to be turned into apoptotic machineries by transducing apoptotic downstream pathways through ATF6, PERK, and IRE1 signaling pathways [19].

## 2. Anti-Cancer Strategy Based on Adaptive Pathway of ER Stress in Cancer Growth

**Figure 1:**
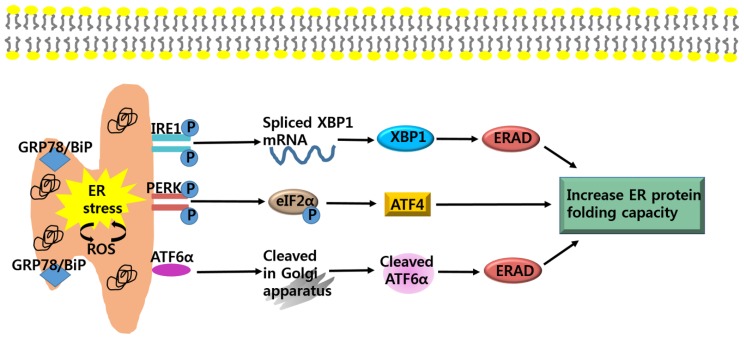
Adaptive pathways of endoplasmic reticulum (ER) stress. ER stress is induced by an accumulation of unfolded proteins in the ER lumen. During ER stress, glucose-regulated protein 78 (GRP78)/ binding protein (BiP) dissociates from its interaction with the three ER stress sensors, inositol-requiring protein 1 (IRE1), protein kinase RNA-like ER kinase (PERK), and activating transcription factor 6 (ATF6), which become activated. IRE1 mediates splicing of α-x-box binding protein 1 (XBP1), which is responsible for the upregulation of ER associated degradation (ERAD). PERK phosphorylates eIF2α and induce activating transcription factor 4 (ATF4), which is involved in restoring ER homeostasis. ATF6α cleaved by specific Golgi resident proteases, increase expression of UPR genes and ERAD. Eukaryotic initiation factor 2 (eIf2); protein kinase RNA-like ER kinase (PERK).

Cancer cells evolve UPR to mitigate the ER stress condition as a survival strategy for progression [20]. Also, UPR in cancer is reported to have significant roles in having resistance to chemotherapy or radiation [17,21]. Therefore, one therapeutic rationale regarding suppression of cancer is to facilitate the accumulation of unfolded proteins by inhibiting components of UPR involved in survival response, ultimately leading to apoptosis [22] (Figure 1). 

### 2.1. Anti-Cancer Strategy; Targeting ATF6α-GRP78/BiP Signaling

A first modulator of ER stress signaling is ATF6α-GRP78/BiP. During ER stress conditions, dissociation of GRP78/BiP from ATF6 allows this protein to translocate to Golgi apparatus where it is cleaved by specific Golgi resident proteases [23]. This process forms ATF6α, which produces active transcription factors that are involved in up-regulating ER chaperones and ERAD [24]. In addition, ATF6α contributes to transcription factor XBP1, which targets the ERAD [25] (Figure 1). Further, the expression of GRP78/BiP is elevated particularly in liver, colon, prostate, breast, lung, and gastric cancer [26,27,28]. The elevated expression of GRP78/BiP is reported to correlate with cancer proliferation, chemotherapy resistance, and poor patient survival rate by modulating accumulation of unfolded protein folding [29,30,31]. In addition, GRP78/BiP induces angiogenesis by activating the potent pro-angiogenic factor, vascular endothelial growth factor A (VEGF-A) [32]. Recently, studies have confirmed that knockdown of GRP78/BiP by siRNA can inhibit carcinogenesis and sensitize cancer cells to chemotherapeutic agents as well as ER stress [33,34,35]. The GRP78/BiP inhibitor exerted cytotoxicity against leukemia [36] and glioma cancer cells [34]. These observations suggest that during ER stress, inhibition of the ATF6-GRP78/BiP signaling pathway could be a strategy for cancer therapy [37]. For example, the knockout of GRP78 resulted in decreased proliferation rates of glioma cells and suppressed PTEN null prostate tumorigenesis and AKT oncogenic pathway [34,38].

### 2.2. Anti-Cancer Strategy; Targeting IRE1α-XBP1 Signaling

A second modulator of ER stress signaling is IRE1α-XBP1. Two isoforms of IRE1, IRE1α and IRE1β, have been identified. IRE1α is expressed in all cell types, but the expression of IRE1β is specifically restricted to the intestinal epithelium [39,40]. In cells undergoing ER stress, activation of IRE1α stimulates an endoribonuclease that splices XBP1 mRNA to produce mature XBP1. XBP1 encodes an active leucine zipper (bZiP) transcriptions factor that generates transcription of genes involved in ERAD components as well as genes involved in redox homeostasis and oxidative stress response (Figure 1) [41,42]. The involvement of the IRE1α-XBP1 signaling in cancer progression has been the subject of many studies. Upregulated XBP1 expression is observed in various human cancers including breast cancer and hepatocellular carcinoma [43]. XBP1-deficient cancer cells showed hypersensitivity to ER stress or hypoxia condition [41]. Moreover, expression of the dominant-negative form of IRE1α or inhibition of XBP1 by siRNA induces reduction in angiogenesis during tumorigenesis and in cancer growth in xenografts model [15,44,45]. Additionally, inhibition of IRE1α-XBP1 enhances apoptosis by down-regulating several genes involved in UPR and generates reactive oxygen species (ROS) in XBP1-deficient cells [15]. These results provide evidences that deactivation of the IRE1α-XBP1 [46] could be a target of anticancer therapy. For instance, IRE1α inhibitor rendered resistant human glioblastoma cells susceptible to oxidative stress [47]. Inhibition of IRE1α decreased endonuclease activity, increasing cytotoxic activity against human multiple myeloma in vitro and in vivo [48].

### 2.3. Anti-Cancer Strategy; Targeting PERK-eIF2α-ATF4 Signaling

A third modulator of ER stress signaling is the PERK-elf2α signaling pathway. Under ER stress conditions, activation of PERK phosphorylates eIF2α at Ser51 [49]. Phosphorylated eIF2α shuts down protein influx into the ER lumen and attenuates mRNA translation, reducing protein synthesis [50,51]. The activation of PERK-eIF2α increases the translation of a number of mRNAs encoding activating transcription factor 4 (ATF4) [52]. ATF4 promotes cell-survival by controlling amino acid biosynthesis and transport function, as well as antioxidant stress responses [53]. PERK-eIF2α-ATF4 signaling pathway is responsible for cancer growth and resistance against curative treatment. The UPR signaling pathway through the PERK-eIF2α-ATF4 signaling pathway also increases tolerance in cancer cells to hypoxic stress [17]. In addition, the PERK-eIF2α-ATF4 signaling pathway in cancer cells mediates the upregulation of VEGF-A transcription [17]. Recently, it has been reported that PERK-deficient cells are hypersensitive to ER stress [54], and inhibition of PERK suppressed cancer cells which are resistant to radiation in vivo [55]. These results suggest that suppression of PERK-eIF2α-ATF4 signaling pathways could be a target of cancer therapy. For example, transformed mouse embryonic fibroblasts from the PERK-deficient animals and HT29 colorectal carcinoma cells with dominant-negative PERK had lower survival rates under hypoxic conditions than wild type control cells [56]. And these cells displayed smaller cancer-formation and increased the level of apoptotic activity in hypoxic areas, compared to wildtype cells [17]. In addition, PERK-deficient cancer cells exhibited declined ability to induce angiogenesis in response to hypoxic stress [57].

## 3. Anti-Cancer Strategy Based on Apoptotic Pathway of ER Stress in Cancer Growth

**Figure 2:**
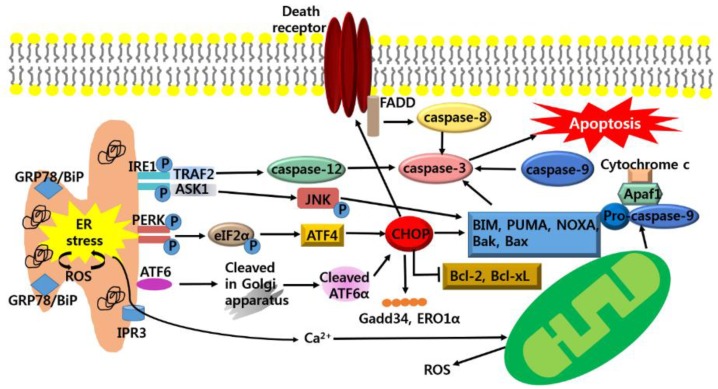
Apoptotic pathways of ER stress. If the adaptive UPR pathway fails to restore homeostasis, chronic ER stress induces apoptosis pathways. ER stress activates IRE1 and activated IRE recruits TNF receptor-associated factor2 (TRAF2) and apoptosis signal-regulating kinase 1 (ASK1). TRAF2 stimulates the activation of caspase-12 and ASK1 elicits the phosphorylation of c-Jun-N terminal kinase (JNK) to activate the pro-apoptotic protein; Bcl-2-like protein 11 (BIM), Bak, Bax and inhibits the anti-apoptotic protein; B-cell lymphoma 2 (Bcl-2), Bcl-2-associated X protein (Bax). Activation of PERK and ATF6 leads to up-regulation of CHOP that regulates Gadd34, ERO1α and activates the pro-apoptotic protein; BIM, Bak, Bax, p53 upregulated modulator of apoptosis (PUMA), NOXA as well as DR which increase caspase-8. Also, ER stress generates ROS and this facilitates the accumulation of unfolded protein in the ER. ER stress-induced Ca^2+^ release into cytosol leads to depolarization of the inner mitochondrial membrane, resulting in Mitochondrial ROS. Apoptosis protease-activating factor 1 (Apaf1); FAS-associated death domain (FADD).

If ER stress persists or is aggravated, cancer cells fail to re-establish ER homeostasis via UPR, ER stress switches from pro-survival to pro-apoptotic condition [58]. Therefore, facilitation of ER stress to initiate the apoptosis pathway could be a therapeutic strategy for anti-cancer activity [14,59] (Figure 2).

### 3.1. Anti-Cancer Strategy; Targeting CHOP-Mediated Apoptosis

During the prolonged ER stress condition, PERK is activated and phosphorylates elF2α [60]. Phosphorylated elF2α subsequently activates ATF4 which targets the expression of apoptotic effector, CHOP (C/EBP-homologous protein; known as GADD153; GENE name Ddit3) [61]. Furthermore, ATF6 is also activated and transcriptionally induces CHOP [62]. CHOP-mediated cell death entails the induction of a variety of genes that may potentiate apoptosis, including GADD34 and ERO1α [63]. GADD34, a transcriptional target of CHOP, encodes a subunit of protein phosphatase that promotes dephosphorylation of eIF2α that leads resumption of protein synthesis, in turn increasing the load of proteins [64]. Accordingly, ER stress activates the UPR to initiate downstream of apoptotic pathways. In addition, CHOP is thought to elevate the expression of Ero1α, which catalyzes the re-oxidation of PDI, leading to hyper-oxidizing conditions in the ER [59]. The CHOP-induced expression of ERO1α activates the ER Ca^2+^ release channel, inositol 1,4,5-triphosphate (IP3) receptor (IP3R) [65]. Then, cytoplasmic Ca^2+^ released from the ER triggers the activation of calcium/calmodulin-dependent protein kinase II (CaMKII), ultimately resulting in the induction of apoptosis pathways [66]. Furthermore, CHOP-mediated apoptosis is involved in extrinsic pathway through up-regulating DR5 along with capase-8 activation, which cleaves Bid (tBid) and translocates it to mitochondria [67,68]. In addition, CHOP is shown to decrease the expression of anti-apoptotic Bcl-2 and Bcl-xL proteins, while increasing the expression of pro-apoptotic proteins including Bak, Bax, BIM (Bcl-2-like protein 11), PUMA (p53 upregulated modulator of apoptosis) and NOXA (PMAIP1) (Figure 2) [69,70]. These studies imply that up-regulation of CHOP expression could be an attractive target for anti-cancer therapy. For example, CHOP-deficiency elevates cancer development in a Kras^G12V^-induced mouse model of lung carcinoma [71]. And hepatocyte-specific CHOP deletion causes cancer growth in a mouse model of hepatocellular carcinoma [72]. 

### 3.2. Anti-Cancer Strategy; Targeting IRE1-Mediated Apoptosis

Under chronic ER stress, IRE1α is induced to activate the mitogen-activated protein (MAP) kinase (MARK) [73]. The IRE1α binds with the adaptor protein, TNF receptor-associated factor2 (TRAF2). IRE1α-TRAF2 complex triggers the activation of caspase-12, which is homologous to human caspase-4 [74]. Caspase-12 translocates from the ER to the cytosol, where it initiates cleavage of procaspase-9, in turn, activating the effector caspase, caspase-3 [75,76]. IRE1α/TRAF2 activates ASK1 (apoptosis signal-regulating kinase 1), which subsequently promotes activation and phosphorylation of c-Jun-N terminal kinase (JNK) [77]. The Phosphorylation of JNK activates BIM, Bak, Bax and inhibits the Bcl-2 and Bcl-xL (Figure 2) [78,79]. These data implicate that the triggering IRE1-mediated apoptosis could be a strategy for anti-cancer therapy. For instance, ER stress-mediated apoptosis was reduced in IRE1-deficient mouse embryonic fibroblasts [80]. And deactivation of ER stress-mediated apoptosis in JNK inhibitor treated wild-type-mouse embryonic fibroblasts was confirmed [81].

### 3.3. Anti-Cancer Strategy; Targeting Generation of ROS-Mediated Apoptosis

When ER stress persists, it hyper-oxidizes the ER lumen, resulting in H_2_O_2_ leakage into the cytoplasm and inducing ROS [82,83]. Furthermore, under oxidative environment, leakage of Ca^2+^ from ER lumen is stimulated, increasing calpains and Ca^2+^ burden in mitochondria [84,85]. The Ca^2+^ uptake in mitochondria depolarizes the inner mitochondrial membrane, leading to mitochondrial ROS generation [86]. Then, BH3-only proteins, Bak and Bax, oligomerize and insert themselves into the outer mitochondrial membrane to increase permeabilization, resulting in the release of cytochrome c [87]. When cytochrome c is released with the formation of APAF-1 (apoptosis protease-activating factor 1) and procaspase-9 [88]; caspase-3 [89], apoptosis-inducing factor (AIF) and endonuclease G (EndoG) are activated [90]. In general, cancer cells intrinsically have more ROS compared to normal cells. Therefore, elevated generation of ROS render cancer cells more susceptible to ER stress, which contributes to ER stress-mediated apoptosis [91]. Thus, the generation of ROS could be a target of anti-cancer therapy. In vitro, ROS-dependent ER stress activation and mitochondrial-related apoptotic pathway were identified in MDA-MB-231 and MCF-7 cell lines with treatment of berberine [92]. Pardaxin-triggered ROS-dependent ER stress and its apoptotic activation via ER stress were inhibited by NAC treatment [93]. 

## 4. Anti-Cancer Effects of Natural Products via ER Stress

The standardized treatments of cancer consist of surgery, chemotherapy, radiation therapy and immunotherapy. These are utilized in accordance with the characters and stages of the cancers. Although the main purpose of anti-cancer treatments is to kill the cancer cells without damaging normal cells, cancer treatments possess limited efficacy and exert their actions on both malignant and normal cells, resulting in adverse effects on patients, including anemia, loss of appetite, delirium and peripheral neuropathy. Thus, the development of effective treatment which has anti-cancer activity with less adverse effects is still needed [94,95]. From the history of drug discovery, natural product-derived compounds could be a promising treatment due to their characteristic to induce apoptosis more commonly in cancers compared to normal cells [96,97]. In fact, several conventional chemotherapeutic agents including Taxol, epothilones, vinca alkaloids originate from the resource of natural products [98]. Currently, as ER stress response is proven to have both an adaptive pathway and apoptotic pathway, modulation of ER stress could be an anti-cancer strategy. Therefore, researchers have focused on the interplay between natural products and anti-cancer effect via ER stress in malignant cells in vitro and in vivo [99,100,101]. Hence, in this review article, anti-cancerous mechanisms of natural products on the most common cancer types via modulation of ER-stress are discussed; lung, breast, colorectal, gastric, prostate, liver cancer, which represent 55% of the global incidence burden in 2012 [2,3].

### 4.1. Search Methodology

Researches regarding the ER stress-mediated apoptotic effect of natural product on cancers were collected from PUBMED/MEDLINE and Google Scholar. Upon searching for appropriate studies, we include ‘ER stress, apoptosis, UPR, natural product, cancers’ for keywords. We collected studies which fit into the criteria: (1) researches based on in vitro or in vivo or clinical trials to prove ER-stress mediated apoptosis of natural product (2); researches that demonstrated reliable statistical analysis data (*p* values that were less than 0.05); and (3) researches that were not upset by subsequent studies. The family names of natural products and herbs mentioned in this review are based on a reliable source. Natural product-derived compounds in this review were double-checked from the NCBI PubChem website for precision.

### 4.2. Natural Products Targeting ER Stress-Mediated Apoptosis in Lung Cancer

Lung cancer is one of the leading causes of cancer-related death in the world and around 1.8 million new cases were estimated in 2012 [3]. Chemotherapy and radiation are the main treatments for lung cancer, which often become resistant to these treatments [102]. As a result, the majority of lung cancer patients suffer from severe side effects and ultimately succumb to death [103]. Therefore, there remains a need for developing a new therapeutic agent to improve the clinical outcomes. As UPR has a critical role in lung cancer viability and lung cancer death [104], researches have been implemented to prove ER stress-mediated anti-cancer activity of natural products against lung cancer cells (Table 1).

Enhanced expression of GRP78/BiP, CHOP, and caspase-3 in NCI-H460 cells were observed by treatment of Polyphyllin D (PD; a potent cytotoxic saponin isolated from *Paris polyphylla*), which suggests that Polyphyllin D-induced ER stress-mediated apoptosis [105]. Dehydrocostuslactone (DHE; sesquiterpene lactone extracted from the *Saussurea lappa* and *Aucklandia lappa*) activated PERK-CHOP and IRE1-JNK signaling pathways in both NCI-H460 and A549 cells through ER stress caused by induction of Ca^2+^ and ROS in the cytoplasm [106]. CHOP expression was upregulated in A549 cells, inducing apoptosis by treatment of brefeldin A (BFA; a macrocyclic lactone antibiotic synthesized from palmitate by a variety of fungi). However, NAC treatment considerably suppressed apoptosis induced by BFA treatment, indicating that BFA induced apoptosis is dependent on ROS [107]. ω-Hydroxyundec-9-enoic acid (ω-HUA; a hydroxyl unsaturated fatty acid derivative isolated from the leaves of *Oryza officinalis*) suppressed the viability of H1299 cells through ROS-dependent ER stress, however, the reduced expression of CHOP was detected after NAC treatment [108]. Curcumin (a phenolic compound isolated from the *Curcuma longa*) elevated the level of CHOP, GRP78/BiP expression in NCI-H460 cells, which is associated with release of intracellular Ca^2+^ [109]. Cantharidin (CTD; a natural terpenoid extracted from *Mylabris phalerata* Pallas) induced cytotoxic effects on NCI-H460 cells through ER stress along with the release of intracellular Ca^2+^, leading to mitochondrial dysfunction. Upregulation of cytochrome c, Bax, Endo G, AIF and cleaved caspase-3,-8 were observed in CTD treated NCI-H460 cells [110]. Also, CHOP expression in A549 and 95-D cells was elevated by furanodiene (FUR; a natural terpenoid isolated from *Curcumae rhizome*) [111]. Parthenolide (PTL; a sesquiterpene lactone derived from the feverfew) treatment upregulated ER stress by activating eIF2α-ATF4-CHOP signaling in A549, Calu-1, H1299, H1792, leading apoptosis. However, knockout of ATF4 by ATF4 siRNA reduced apoptosis, indicating that the expression of CHOP mediates PTL induced-apoptosis [112]. Anacardic acid (AA; a constituent of *Anacardium occidentale*) increased expression of CHOP, cleavage of caspase-12, and disturbed Ca^2+^ homeostasis in A549 cells, confirming AA as an ER stress inducer [113].

### 4.3. Natural Products Targeting ER Stress-Mediated Apoptosis in Breast Cancer

Female breast cancer is the most common malignancy in women and the most frequently diagnosed cancer globally. 1.67 million cases were estimated in 2012, ranking as the second most common cancer world-wide [3]. Until now, most of the therapeutic drugs for breast cancer target the early stage of cancer cells; still there is no available therapeutic options for advanced breast cancer [103]. Thus, the development of more effective anti-cancer agents for breast cancer is imperative. Since the UPR is involved in execution of pro-survival or pro-death decision [114], many studies have been performed to test ER stress-mediated anti-cancer activity of natural products using breast cancer cell lines [115] (Table 2)*.*

BFA, isolated from the fungus *Eupenicillium brefeldianum*, upregulated PERK, CHOP, BIM and downregulated Bcl-2 in MDA-MB-231 cells. These results suggest that mitochondrial dysfunction is involved in BFA-induced ER stress-mediated apoptosis [116]. Activation of elF2α, GRP94, GRP78/BiP, and CHOP in MCF7 cells was demonstrated by cryptotanshinone (a constituent of *Salvia miltiorrhiza* Bunge) treatment. Moreover, cryptotanshinone-induced ER stress sensitized MCF7 cells to anti-cancer agents including TNFα, cisplatin, etoposide and 5-FU [117]. Saxifragifolin D (SD; an oleanane type pentacyclic triterpenoid isolated from *Androsace umbellate*) increased expression of CHOP, JNK and cytochrome c in both MCF-7 and MDA-MB-231 cells through induction of Ca^2+^ and ROS in the cytoplasm, resulting in induction of ER stress [118]. Prodigiosin (a bacterial tripyrrole red-colored pigment produced by *Serratia marcescens*) was proven to have potent cytotoxicity against MCF7, MDA-MB-231 and T-47D cells through ER stress in terms of CHOP expression. Treatment of prodigiosin up-regulated CHOP, cleavage of PARP and decreased Bcl-2. However, decreased cleavage of PARP and increased Bcl-2 were noted after CHOP siRNA treatment, indicating CHOP-dependent apoptosis [119]. Fucoidan (a polysaccharide extracted from brown seaweed such as *Cladosiphon okamuranus* and *Fucus evanescens*) facilitated ER stress by activating eIF2α, CHOP along with suppressing IRE1, XBP1 in MDA-MB-231 cells. Additionally, Fucoidan triggered mitochondrial dysfunction, resulting in Bax and cleavage of caspase-3 [120]. Tocotrienols (a subgroup of vitamine E) promoted increased of CHOP, JNK, cleavage of caspase-3,-8 in MDA-MB-231 and MCF-7 cells. These results indicated that ER stress by Tocotrienols treatment is associated with mitochondria and DR5-mediated apoptosis [101]. Ethanol extract of Brazilian red propolis was reported to activate apoptosis against MCF-7 cells via facilitating ER stress and mitochondria-mediated apoptosis, resulting in CHOP expression, cleavage of caspase-3, Bax, and downregulation of Bcl-xL, Bcl-2 [121]. Expression of CHOP, PERK and ROS was increased in MDA-MB-231 and MCF-7 cells by treatment of ampelopsin (AMP; a main bioactive constituent of *Ampelopsis grossedentata*). However, NAC treatment triggered inhibition of CHOP expression, indicating that AMP-induced ER stress-mediated apoptosis is dependent on ROS [122].

### 4.4. Natural Products Targeting ER Stress-Mediated Apoptosis in Colorectal Cancer

Colorectal cancer is one of the leading causes of malignant neoplasm-related death in the world [3]. Around 1.3 million new cases were estimated, ranking as the third most common cancer in the world. Although early stage colon cancer is treatable, it is known to be resistant to chemotherapeutic drugs, which is linked with poor patient prognosis [123]. As UPR is involved in regulating colorectal cancer cell fates [124], many researches have been performed to test ER stress-mediated anti-cancer potential effects of natural products using colorectal cancer cells (Table 3). 

Curcumin enhanced expression of CHOP, JNK, cytochrome c, and FADD in HT-29 cells. These results indicate that the release of intracellular Ca^2+^, mitochondrial dysfunction and DR5 are associated with curcumin-induced ER stress-mediated apoptosis [125]. 2-(3,4 dihydroxyphenylethanol) ethanol (DPE; phenol antioxidant derived from olive oil) induced apoptosis on HT-29 cells via ER stress, activating PERK-eIF2α-CHOP and IRE1-JNK signaling pathway. Moreover, mitochondria-mediated apoptotic factors including Bax, Bad, cytochrome c, and cleavage of caspase 3 were observed [126]. Suppression of Colo 205 cells viability was detected via BFA-induced ER stress in terms of CHOP expression [127]. Activation of eIF2α, CHOP cleavage of caspase-4 was upregulated in HT29 cells by treatment of resveratrol (natural polyphenolic compound) [128]. Expression of PERK-ATF4-CHOP signaling was activated by treatment of zerumbone (ZER; sesquiterpene purified isolated from the *Zingiber zerumbet* Smith). Also, ZER up-regulated DR5 expression in both HCT116-p53null cells and SW480 cells, eventually activating caspase-8 [129]. Guttiferone H (derived from *Garcinia xanthochymus*) elevated expression of CHOP along with cleavage of caspases-3,-7 in HCT116 cells [130]. Inhibition of HCT116 cells viability following fucoidan (derived from *Cladosiphon okamuranus* and *Fucus evanescens*) treatment was observed, which is induced by upregulation of elF2α-CHOP expression along with inhibition of IRE1-XBP1 [120]. Piperine (from *Piper nigrum* Linn and *Piper longum* Linn) generated ROS, CHOP, JNK, cytochrome c in HT-29 cells. These findings indicate that ER stress-mediated apoptosis by piperine is linked with mitochondrial dysfunction [131]. Potent anti-cancer activity of flavokawain B (derived from *Alpinia pricei* Hayata) against HCT116 cells was proven through ER stress in terms of CHOP expression. However, NAC treatment restored the inhibitory effects of Flavokawain B, suggesting that ROS generation is required for ER stress-mediated apoptosis [132].

### 4.5. Natural Product Targeting ER Stress-Mediated Apoptosis in Gastric Cancer

Gastric cancer emerged as one of the leading neoplasms in the world. Almost 1 million new cases of stomach cancer were estimated to have occurred in 2012, making it the fourth most common cancer world-wide [3]. Although progresses in surgery and chemotherapy for gastric cancer have been achieved, there is a high rate of cancer recurrences [133]. Therefore, novel effective therapeutic agents need to be developed. As UPR have a regulatory mechanism in either pro-survival or pro-apoptotic signals in gastric cancer [134], current studies have been performed to demonstrate ER-mediated anti-cancer activity of natural products (Table 4). 

Curcumin enhanced CHOP, JNK, cytochrome c, FADD, DR5 and caspase-8 in AGS cells. These results indicate that the release of intracellular Ca^2+^, mitochondrial dysfunction, extrinsic apoptotic pathway are involved in curcumin-induced ER stress [125]. Ultrafine (AM2; ethanolic extract of pulverized particles from *Ulmus davidiana var.* Japonica) upregulated GRP78/BiP, p-eIF2α, cleavage caspase-3,-6,-9 in both SNU-1 and SNU-484 cells [135]. Inhibition of MKN45 and SCM-1 cells viability through CHOP expression along with reduction of GRP94 was detected in Honokiol (isolated from the *Magnolia officinalis*) treatment [136]. Vitamin E succinate (RRR-a-tocopheryl succinate, VES) increased the level of CHOP, JNK, and caspase-12 and downregulated the expression of GRP94 in SGC-7901 cells [137]. Activation of CHOP, JNK and cleavage of caspase-4 were upregulated in SGC-7901 cells after treatment with a-Tocopheryl succinate (a-TOS; a derivative of natural vitamin E). NAC treatment decreased cell death by a-TOS, indicating that ER stress-mediated apoptosis by a-TOS is dependent on ROS generation [138]. Casticin (a major component obtained from *Fructus viticis*) induced elevation of CHOP, eIF2α, GRP78/BiP in BGC-823 cells as ER stress inducer. Additionally, Casticin upregulated DR5, leading to caspase-8 cleavage [139]. Expression of eIF2α-ATF4-CHOP signaling and JNK were enhanced in SGC-7901 cells by treatment of WZ35 (mono-carbonyl analogs of curcumin (MACs) via deletion of β-diketone moiety). Moreover, mitochondria-mediated apoptotic factor including Bax; cleavage of caspase-3 was observed. However, NAC treatment inhibited the effect of WZ35 by downregulating eIF2α-ATF4-CHOP signaling, Bax and cleavage of caspase-3. These results suggest that ROS generation may interplay with its ER stress-mediated apoptosis [140].

### 4.6. Natural Products Targeting ER Stress-Mediated Apoptosis in Prostate Cancer

Prostate cancer is the second most common cancer in men, and an estimated 1.1 million cases were diagnosed worldwide in 2012, making it the fourth leading cancer [3]. Due to early detection and new treatment options, quality of life of prostate cancer patients has been improved. However, most of the patients with prostate cancer suffer from aggressive and refractory cancer, with very poor prognosis [141]. Since UPR has been demonstrated to have an influence on either pro-survival or pro-death processes in prostate cancer [142]; currently, many studies have been reported to discover natural product with ER stress-mediated anti-cancer activity to develop novel therapeutic agents (Table 5). 

Ardisianone (from *Ardisia virens* Kurz) elevated the level of GRP78/BiP, cytochrome c, AIF, generation of ROS and cleavage of caspases-3,-9 in PC-3 cells, indicating that ER stress and mitochondria-mediated apoptosis were related to ardisianone treatment [143]. Induction of apoptosis in PNT1a and PC3 cells through CHOP expression, triggering BH3-only proteins, AIF and cleavage of caspase-3,-9 was demonstrated by the treatment of Polyphenol E (derived from *Camellia sinensis*) [144]. Mangosteen Fruit Extract (MFE; derived from *Garcinia mangostana*) enhanced the level of PERK, CHOP, IRE1 and caspase-4 in both LNCaP and 22Rv1 cells through ER stress, leading to Bax and cleavage of caspase-3 [145]. Upregulation of eIF2α-ATF4-CHOP signaling and caspase-4 in PC-3, DU145, and LNCaP cells through ER stress was observed after treatment of Marchantin M (Mar; derived from *Astrella angusta*) [146]. Monascuspiloin (derived from *Monascus pilosus*) treatment facilitated ER stress by activating eIF2α-ATF4-CHOP signaling pathway and caspase-3,-4 in PC-3 cells, which renders PC-3 cells sensitive to ionizing radiation [147]. Quercetin, a ubiquitous polyphenol found in several plants, increased the level of GRP78/BiP, ATF4, IRE1 and caspase-12 in PC-3 cells through ER-stress, which is associated with the induction of Ca^2+^ and ROS in the cytoplasm. Activation of Bax, cytochrome c, AIF, Endo G, and caspase-3,-9 were also detected [148]. Elevated levels of GRP78/BiP and CHOP in PC-3 cells through ER stress were induced by ZER treatment, resulting in activating cleavage of caspase-3,-7,-9, PARP and Bid along with inhibition of Bcl-2 [149].

### 4.7. Natural Product ER Stress-Mediated Apoptosis in Liver Cancer

Liver cancer emerged as one of the leading malignancy-related deaths globally. About 7.8 million new cases occurred in 2012, making it the sixth leading malignant neoplasm in the world [3]. Surgery is the main curative treatment for liver cancer, but a majority of patients with liver cancer are diagnosed at advanced stages. Thus chemotherapy has remained the ultimate curative approach for treatment of liver cancer [150]. However, patients tend to suffer from adverse side effect since chemotherapy agents kill both cancer cells and normal cells [151]. ER stress-mediated anti-proliferative effects of natural product have been elucidated through many studies using liver cancer cells [152] (Table 6).

Licochalcone A (derived from *Glycyrrhiza inflate*) elevated the expression of GRP78/BiP, ATF6, IRE1α, caspase-4, and CHOP in HepG2 cells, leading to cleavage of caspases-3,-9. However, ER stress-mediated apoptosis was inhibited by NAC treatment, indicating ROS involvement in Licochalcone A-induced ER stress [153]. Guggulsterone (originated from *Commiphora mukul*) increased the levels of GRP78/BiP, PERK, CHOP, JNK, DR5 in Hep3B cells through ER stress. Knockout of CHOP by CHOP siRNA reduced DR5 expression and NAC reverted a suppressive effect of guggulsterone, indicating CHOP-dependent DR5 and ROS-dependent ER-stress by guggulsterone [154]. Enhancement of CHOP-induced DR5 in Hep3B and HepG2 was noted after treatment of verrucarin A (derived from *Fungal metabolite*) [155]. 7-dimethoxyflavone (DMF; extracted from *Myrtacea L. scoparium* Forst and *Piper methysticum* Forst, etc) upregulated the level of ATF4, CHOP, DR5, cleavage of caspase-3,-8,-9 in Hep3B cells. This result suggests that both intrinsic and extrinsic apoptosis pathways are associated with DMF-induced ER stress [156]. GRP78/BiP, PDI, cleavage of caspase-3 and BH-3 family including BIM Bid were upregulated in Hep3B cells by treatment of neferine (isolated from *Nelumbo nucifera* Gaertn), indicating that ER stress and mitochondria-mediated apoptosis were induced by neferine [157]. Paenol (Pae; derived from *Pycnostelma paniculatum* K. Schum) elevated the level of GRP78/BiP, CHOP and cleavage of caspase-3 in HepG2 cells. This attenuated ER stress-induced resistance of HepG2 to the therapeutic agent doxorubicin [158]. Expression of elF2α, CHOP, JNK, MAPK in HepG2 cells were elevated through ER stress following cryptotanshinone treatment. However, NAC treatment down-regulated the apoptotic effect of cryptotanshinone. This result indicates that ER stress-mediated apoptosis via Cryptotanshinone is dependent on ROS generation [117]. 6-Shogaol (extracted from *Zingiber officinale* Rosc) inhibited SMMC-7721 cells growth via ER stress in terms of PERK-eIF2α-CHOP expression. In addition, oral administration of 6-Shogao suppressed the pro-survival pathway of p-PERK, eIF2a and p-eIF2α while it increased the cleavage of caspase-3, leading to reduction in SMMC-7721 tumor xenografts model [159]. With the induction of elevated CHOP, caspase-12 expression in Hep3B cells were noted after treatment of genistein (a flavonoid derived from soy products). In addition, activation of Apaf-1, cytochrome c, and cleavage of caspase-3,-9, PARP were observed, indicating mitochondria-mediated apoptosis [160].

## 5. Discussion

Cancer remains one of the leading causes of death in the world today. Although significant medical and technological developments have been achieved, still conventional cancer-targeted therapies have severe side effects and complications such as serious toxicities and development of resistance [161,162]. Therefore, one of the major goals for cancer treatment is to find novel therapeutic approaches which make it possible to selectively kill cancer cells without harming normal cells and reduce cancer cellular resistance to chemotherapeutic agents [145,147,158,163].

Upon quick expansion of the cancer tumor-microenvironment such as hypoxia, lack of glucose may have an impact on protein folding in ER of cancer cells, resulting in the accumulation of unfolded proteins, termed ER stress [22]. To overcome the hostile environment, UPR is initiated in cancer cells to attenuate ER stress for cancer survival. In fact, proteins related to UPR such as GRP78/BiP, XBP1, IRE1 were reported to be promoted in ischemic regions of cancer [17]. Also, activation of GPR78/BiP, XBP1 and ATF6 were observed in hepatocellular carcinoma cell line compared to non-cancerous liver tissues [164]. In addition, ER stress response may generate drug resistance as a survival response via activation of UPR [165]. For example, induction of the UPR in multiple myeloma cells enhanced resistance to etoposide [166]. These indicate that ER stress response may act as a key driver in tumorigenesis and the development of resistance to chemotherapy [43]. However, if the ER stress persists, apoptosis is induced in cancer cells [167]. These related findings provide clues that targeting the ER stress response, either inhibiting adaptive function or stimulating apoptotic function, may be an effective strategy for more selective cancer therapy and overcoming drug resistance [147]. 

As a way of cancer treatment, natural product-derived compounds are being introduced to the medical research and conducted to test the efficacy against cancers [168,169]. Especially, researches focusing on the cross link between the natural product or its derived compound and apoptosis in cancer via ER stress, are gaining interest [170,171]. Natural products or their bioactive compounds not only trigger apoptosis but also lower the resistance to chemotherapies via modulating ER stress. For example, brefeldin A derived from *Eupenicillium brefeldianum* was proven as an ER stress-inducing agent to overcome one of the standard drugs for leukemia fludarabine [172]. Also, Pae, which is derived from *Pycnostelma paniculatum* K. Schum, lowers the resistance of doxorubicin [158]. Furthermore, natural compounds were demonstrated to induce a synergistic effect with current standard therapies through ER stress regulation. For instance, Epigallocatechin 3-gallate, a polyphenolic green tea component, increased the apoptotic activity of temozolomide in glioblastoma cell death through inhibition of GPR78/BiP [173]. And falcarindiol, a natural polyyne in dietary plants, was identified to induce a synergistic effect with 5-Fluorouracil and bortezomib on suppressing breast cancer via upregulation of CHOP [174]. 

This present review summarizes the mechanisms of ER stress and its role in cancer development as well as apoptosis. It also delineates two approaches targeting the UPR as an anticancer strategy [19]: To inhibit ER stress–related proteins, to render cancer growing under stressful conditions to no longer be able to deal with the stress [11], and to induce chronic stress on cancer cells thereby tipping the balance towards cell death. Furthermore, the up-to-date researches were highlighted which were done with natural products in relation to the ER stress-mediated apoptosis in six types of leading cancers world-wide: lung, breast, colorectal, gastric, prostate cancer, and liver cancer, referring to the World Cancer Report 2014 by International Agency for Research on Cancer [2,3]. The related studies in this topic will not only significantly clarify our understanding of ER stress in cancer biology but also advance knowledge of interplay between natural product and ER stress-mediated apoptosis against cancer. Although cumulative evidences demonstrate that targeting ER stress as an anti-cancer strategy seems very promising. Further studies for the exact roles of ER stress in cancer and how they act on cell fate need to be investigated. Increasing knowledge in this area will be essential for pharmaceutical design toward controlling cancer through modulating UPR signaling [20,33]. Moreover, further research into ER stress could be utilized for other medical applications including the prognosis and diagnosis of multiple cancer types and could also be a solution to its involvement in chemo-resistance [26,35]. 

However, to successfully convert a potent natural product-derived compound to a clinically viable drug, several following questions needs to be addressed: (i) Do natural products exert selective cytotoxicity on cancer cells not on normal cells? (ii) Is it effective to target the ER stress as an anti-cancer strategy against other types of cancers? (iii) Do natural products or their derived compounds possess any special effects compared to those often seen with conventional drugs for cancer treatment? (iv) How might natural products cooperate with therapeutic agents that cause cancer cellular stress? (v) Can natural products or their derivatives induce a synergistic effect with conventional cancer treatment or unexpected side effects? Furthermore, since natural products or their molecular approaches targeting ER stress in cancer treatment are only in the early stage of research, most of the studies have focused on in vitro. Hence, additional investigation into pharmacokinetics with animal models and clinical studies are necessary. In addition, proper dosage of natural product needs to be considered to prevent the potential toxicity produced by natural products when developing natural product-derived compounds into clinically viable drugs [175]. For instance, ZER, which induces ER stress-mediated apoptosis in prostate cancer, was demonstrated to cause nephrocellular and hepatocellular damage by single intraperitoneal injection of 500 mg/kg ZER in sprague dawley (SD) rats [176]. Polyphenon E, which suppresses prostate cancer growth through ER stress, was identified to induce the mutant frequency in thymidine kinase locus and damage the myocardial fiber of Big Blue transgenic mouse when administered at a dosage of 2000 mg/kg for 28 days [177].

Eventually, more researches in this area will make clear understanding about the underlying mechanism of cross link between natural products and ER mediated-apoptosis. Also, enhanced knowledge in this area can be impetus for future developments of novel medicine not only in cancer treatment but also other diseases known to be associated with ER stress such as cardiovascular, inflammatory, and neurological diseases [178,179]. Importantly, additional evaluation of the therapeutic effects of natural products on cancer would provide us with an effective strategy to potentiate the health benefits of natural products.

## Figures and Tables

**Table 1:** Bioactive compounds from natural products that induce ER-stress-mediated apoptosis in lung cancer.

Family Name	Compound	Cell Line	Duration/Dosage	Mechanism	Reference
*Paris polyphylla*	Polyphyllin D	NCI-H460	1.8 µM, 8 h	CHOP ↑, GRP78/BiP ↑, PDI ↑	[105]
				cleavage of caspase-3,-4,-9,-12 ↑, Bax ↑, Bcl-2 ↓	
*Saussurea lappa* and *Aucklandia lappa*	Dehydrocostuslactone	NCI-H460	15 μg/mL, 1 h	release of intracellular Ca^2+^ levels ↑, Δψm ↓, p-PERK ↑, GRP78/BiP ↑, IRE1 ↑, CHOP ↑, XBP-1 ↑	[106]
				Cleavage of caspase-4 ↑, JNK ↑, MAPK ↑	
				ROS ↑	
		A549	15 μg/mL, 1 h	release of intracellular Ca^2+^ ↑, Δψm ↓,p-PERK ↑, GRP78/BiP ↑, IRE1 ↑, CHOP ↑, XBP1 ↑	
				JNK ↑, MAPK ↑, cleavage of caspase-4 ↑	
				ROS ↑	
	BrefeldinA	A549	1 µM, 36 h	p-PERK ↑, IRE1α ↑, ATF4 ↑, ATF6 ↑, CHOP ↑, GRP78/BiP ↑	[107]
				cleavage of PARP ↑, cleavage of caspase-2 ↑, Bid ↑	
*Oryza officinalis*	ω-Hydroxyundec-9-enoic acid	H1299	500 µM, 24 h	p-eIF2α ↑, CHOP ↑	[108]
				cleavage of caspase-6,-9 ↑, cleavage of PARP ↑	
				ROS ↑	
*Curcuma longa*	Curcumin	NCI-H460	25 µM, 24 h	release of intracellular Ca^2+^ ↑, Δψm ↓, CHOP ↑, GRP78/BiP ↑	[109]
				Bcl-2 ↓, Bcl-xL ↓, cytochrome c ↑, cleavage of caspase-3,-8,-9 ↑, *BAX* ↑, *BAD* ↑	
				ROS ↑	
*Mylabris phalerata* Pallas	Cantharidin	H460	10 µM, 24 h	release of intracellular Ca^2+^ ↑, Δψm ↓, GRP78/BiP ↑, IRE1α ↑, IRE1β ↑, ATF6α ↑, XBP1 ↑, calpain ↑,	[110]
				Bcl-xL ↓, cleavage of caspase-3,-8,-4 ↑ cytochrome c ↑ *BAX* ↑, AIF ↑, Endo G ↑	
				ROS ↑	
*Curcumae Rhizoma*	Furanodiene	A549, 95-D	80 µM, 24 h	CHOP ↑, BIP ↑	[111]
*Tanacetum parthenium* L.	Parthenolide	A549, Calu-1, H1299, H1792	20 µM, 24 h	ATF4 ↑, p-eIF2a ↑, eIF2α ↑	[112]
				cleavage of caspase-3,-8,-9 ↑, cleavage of PARP ↑	
*Anacardium occidentale*	Anacardic acid	A549	3.0 μg/mL, 18 h	release of intracellular Ca^2+^ ↑, Δψm ↓, GRP78/BiP ↑, CHOP ↑, IRE1α ↑ ATF6 ↑, p-PERK ↓, p-eIF2α ↓	[113]
				cleavage caspase-12 ↑	

Glucose-regulated protein 78 (GRP78); binding protein (BiP); protein disulphide isomerase (PDI); protein kinase RNA-like ER kinase (PERK); reactive oxygen species (ROS); inositol-requiring protein 1 (IRE1); ↑ - increasing concentration; ↓ - decreasing concentration; activating transcription factor 4 (ATF4); activating transcription factor 6 (ATF6); B-cell lymphoma 2 (Bcl-2), Bcl-2-associated X protein (Bax); X-ox binding protein 1 (XBP-1); c-Jun-N terminal kinase (JNK); mitochondrial membrane potential (Δψm); C/EBP-homologous protein (CHOP); α-x-box binding protein 1 (XBP1); apoptosis-inducing factor (AIF); and eukaryotic initiation factor 2 (eIf2).

**Table 2:** Bioactive compounds from natural products that induce ER stress-mediated apoptosis in breast cancer.

Family Name	Compound	Cell Line	Duration/Dosage	Mechanism	Reference
*Eupenicillium brefeldianum*	Brefeldin A	MDA-MB-231	1 μg/mL, 24 h	IRE1α ↑, PERK ↑, CHOP ↑, calnexin ↓	[116]
				BIM ↑, cleavage of PARP ↑, Bcl-2 ↓,	
*Salvia miltiorrhiza* Bunge	Cryptotanshinone	MCF7	10 µM, 24 h	p-eIF2α ↑, GRP94 ↑, GRP78 ↑, CHOP ↑	[117]
				cleavage of PARP ↑, cleavage of caspase3 ↓	
				ROS ↑	
*Androsace umbellata*	Saxifragifolin	MDA-MB-231	5 µM, 24 h	IRE1α ↑, calnexin ↑, calpain ↑, XBP1 ↑, CHOP ↑, GRP78/BiP ↑,	[118]
				cleavage of PARP ↑, cleavage of caspase-3,-9 ↑, Bax ↑, cytochrome C ↑, p-JNK ↑	
				ROS ↑	
		MCF7	5 µM, 24 h	IRE1α ↑, Calnexin ↑, calpain ↑, XBP-1 ↑, GRP78/BiP ↑, CHOP ↑	
				cytochrome c ↑	
*Serratia marcescens.*	Prodigiosin	MCF7 MDA-MB-231T-47D	100 µM, 24 h	GRP78 ↑, CHOP ↑, p-IRE1 ↑, IRE1 ↑, p-eIF2a ↑, eIF2a ↑, ATF6 ↑	[119]
				cleavage of PARP ↑, p-JNK ↑, JNK ↑, BCL-2 ↓	
*Fucus vesiculosus*	Fucoidan	MDA-MB-231	100 μg/mL, 72 h	CHOP ↑, ATF4 ↑, p-eIF2α ↑, GRP78/BiP ↓, p-IRE1 ↓, XBP1 ↓	[120]
				Bax ↑, CaMK II ↑, cleavage of caspase-3,-12 ↑, cleavage of PARP ↑	
	γ-tocotrienol	MDA-MB-231	40 µM, 16 h	CHOP ↑, ATF4 ↑, GRP78/BiP ↑, XBP1 ↑	[101]
				cleavage of PARP ↑, cleavage of caspase-3,-8,-9 ↑, DR5 ↑, JNK ↑, p-JNK, C-Jun ↑, p38 MAPK ↑	
		MCF-7	40 µM, 16 h	CHOP ↑, GRP78/BiP ↑, XBP1 ↑	
				cleavage of PARP ↑, cleavage of caspase-8,-9 ↑, JNK ↑, p-JNK ↑, C-Jun ↑, DR5 ↑, p38 MAPK ↑	
Brazilian Red propolis	Ethanol extract of Brazilian Red propolis	MCF-7	20 μg/mL, 24 h	CHOP ↑	[121]
				cleavage of caspase-3 ↑, Bax ↑, BcL-xL ↓, BcL-2 ↓	
*Ampelopsis grossedentata*	Ampelopsin	MCF-7 MDA-MB-231	60 µM, 24 h	GRP78/BiP ↑, p-PERK ↑, p-eIF2α ↑, ATF6 ↑, CHOP ↑	[122]
				ROS ↑	

Bcl-2-like protein 11 (BIM); death receptor (DR5).

**Table 3:** Bioactive compounds from natural products that induce ER stress-mediated apoptosis in colorectal cancer.

Family Name	Compound	Cell Line	Duration/Dosage	Mechanism	Reference
*Curcuma longa*	Curcumin	HT-29	40 μM, 24 h	release of intracellular Ca^2+^, Δψm ↓, CHOP ↑	[125]
				cleavage of caspase-3,-7,-8,-9 ↑, Fas ↑, FasL ↑ FADD ↑ Bid ↑, tBid ↑, p-JNK ↑, Bax ↑, cytochrome c ↑, Bcl-2 ↓	
*Olea europaea*	2-3,4 dihydroxyphenylethanol	HT-29	400 μM, 16 h,	release of intracellular Ca^2+^ ↑, Δψm ↓, IRE1 ↑, XBP1 ↑, GRP78/BiP ↑, PERK ↑, eIF2α ↑, CHOP ↑,	[126]
				Bax ↑, Bak ↑, Bad ↑, cytochrome c ↑, cleavage of caspase 3 ↑, TRAF2 ↑, ASK ↑, JNK ↑, AP-1 ↑, p-JNK ↑ C-jun ↑, PI3K/Akt ↓, Bcl-2 ↓	
*Eupenicillium brefeldianum*	Brefeldin A (BFA)	Colo 205	15 ng/mL, 24 h	GRP78 ↑, XBP1 ↑, CHOP ↑	[127]
	Resveratrol	HT29	50 μM, 24 h	GRP78/BiP ↑, CHOP ↑, XBP1 ↑, eIF2α ↑	[128]
				cleavage of caspase-4 ↑, cleavage of PARP ↑	
*Zingiber zerumbet* Smith	Zerumbone	HCT116-p53null	20 μM, 24 h	ATF4 ↑, CHOP ↑, GRP78/BiP ↑, p-PERK ↑, PERK ↑ eIF2α ↑, p-eIF2α ↑	[129]
				DR5 ↑	
				ROS ↑	
		SW480	20 μM, 24 h	ATF4 ↑, CHOP ↑, GRP78/BiP ↑, p-PERK ↑, PERK ↑ eIF2α ↑, p-eIF2α ↑	
				DR5 ↑	
				ROS ↑	
*Garcinia xanthochymus*	Guttiferone H	HCT116	10 μg/mL, 24 h	ATF4 ↑, XBP1 ↑, CHOP ↑	[130]
				Cleavage of caspases-3,-7 ↑	
*Cladosiphon okamuranus* and *Fucus evanescens*	Fucoidan	HCT116	100 μg/mL, 72 h	GRP78↑, p-CaMKII ↑, eIF2a ↑, p-eIF2α ↑, CHOP ↑, IRE1 ↓, XBP1 ↓	[120]
				Cleavage of PARP ↑	
*Piper nigrum* Linn and *Piper longum* Linn	Piperine	HT-29	100 μg/mL, 72 h	IRE1α ↑, CHOP ↑, GPR78/BiP ↑	[131]
				cleavage of PARP ↑, cytochrome c ↑, JNK ↑, MAPK ↑, PI3K/Akt ↓	
				ROS ↑	
*Alpinia pricei* Hayata	Flavokawain B	HCT116	50 μM, 8 h	CHOP ↑, ATF4 ↑	[132]
				Bcl-2 ↓, MAPK ↑, PARP ↑, cytochrome c ↑, BIM ↑, Bak ↑	
				ROS ↑	

TNF receptor-associated factor2 (TRAF2); apoptosis signal-regulating kinase 1 (ASK1); calcium/calmodulin-dependent protein kinase II (CaMKII); Fas ligand (FasL).

**Table 4:** Bioactive compounds from natural products that induce ER stress-mediated apoptosis in gastric cancer.

Family Name	Compound	Cell Line	Duration/Dosage	Mechanism	Reference
*Curcuma longa*	Curcumin	AGS	20 µM, 24 h	Release of intracellular Ca^2+^ ↑, Δψm ↓, CHOP ↑	[125]
				cleavage of caspase-3,-7,-8,-9 ↑, cytochrome c ↑	
*Ulmus davidiana var.* japonica	Ultrafine	SNU-1	200 μg/mL, 24 h	GRP78/BiP ↑, p-eIF2α ↑	[135]
				cleavage caspase-3,-6,-9 ↑, cleavage of PARP ↑	
		SNU-484	300 μg/mL, 24 h	p-eIF2α ↑, GRP78/BiP ↑	
				cleavage of caspase-3,-6,-9 ↑, cleavage of PARP ↑, Bcl-2 ↓, Bcl-xL ↓	
*Magnolia officinalis*	Honokiol	MKN45	40 µM, 8 h	GRP94 ↓, CHOP ↑, calpain ↑	[136]
				cleavage of caspase-7,-12 ↑, cleavage of PARP ↑	
		SCM-1	40 µM, 24 h	GRP94 ↓, CHOP ↑, calpain ↑	
				cleavage of PARP ↑,	
vitamin E	Vitamin E succinate	SGC-7901	20 μg/mL, 24 h	release of Intracellular Ca^2+^ ↑, Δψm ↓ GRP78/BiP ↑, GRP94 ↓, PERK ↑, ATF4 ↑, ATF6 ↑, XBP1 ↑, CHOP ↑	[137]
				cleavage of caspase-4,-7,-12 ↑, p-JNK ↑, JNK ↑, cleavage of PARP ↑	
vitamin E	A-tocopheryl succinate	SGC-7901	20 μg/mL, 24 h	GRP78/BiP ↑, CHOP ↑	[138]
				cleavage of caspase-4 ↑	
				ROS ↑	
*Fructus viticis*	Casticin	BGC-823	1 μmol/mL, 24 h	CHOP ↑, p-eIF2α ↑, eIF2α ↑, GRP78/BiP ↑	[139]
				DR5 ↑, Bax ↑, Bid ↑, cleavage of caspase-3,-8,-9 ↑	
				ROS ↑	
*Curcuma longa*	1-(4-hydroxy-3-methoxyphenyl)-5-(2-nitrophenyl)penta-1,4-dien-3-one (WZ35)	SGC-7901	10 μM, 12 h	ATF6 ↑, ATF4 ↑, XBP1 ↑, CHOP ↑	[140]
				P-JNK ↑, Bax ↑, cleavage of caspase-3 ↑ Bcl-2 ↓	
				ROS ↑	
		SGC-7901xenograft in athymic BALB/Ca-nu/nu female mice	WZ35 (orally, 50 mg/kg) for 28 days	CHOP ↑	
				cleavage of caspase-3 ↑	

**Table 5:** Bioactive compounds from natural products that induce ER stress-mediated apoptosis in prostate cancer.

Family Name	Compound	Cell Line	Duration/Dosage	Mechanism	Reference
*Ardisia virens* Kurz	Ardisianone	PC-3	10 μg/mL, 24 h	GRP78/BiP ↑	[143]
				cleavage ofcaspases-3,-7,-8,-9 ↑, cleavage of PARP ↑, Bcl-2 ↑, Bcl-xL ↑, Bak ↑, Bax ↑, Bid ↑, PI3K/Akt ↓, cytochrome c ↑, AIF ↑	
				ROS ↑	
*Camellia sinensis*	Polyphenon E	PNT1a	35 μg/mL, 12 h	ATF4 ↑, PERK ↑, p-eIF2α ↑, eIF2α ↑, GRP78/BiP ↑, CHOP ↑, XBP1 ↑	[144]
				cleavage of caspase-3,-7,-9 ↑, Bak ↑, Puma ↑, cleavage of PARP ↑	
				ROS ↑	
		PC3	145 μg/mL, 12 h	ATF4 ↑, PERK ↑, p-eIF2α ↑, eIF2α ↑, CHOP ↑, XBP1 ↑,	
				cleavage of caspase-3,-7,-9 ↑, Puma ↑, Bak ↑, Bax ↑, PARP ↑ AIF ↑	
				ROS ↑	
*Garcinia mangostana*	Mangosteen Fruit Extract	LNCaP	15 μg/mL, 24 h	PERK ↑, IRE1 ↑, CHOP ↑, GRP78/BiP ↑, Ero1 ↑, ER chaperone ↑, PDI ↑, XBP1 ↑, calnexin ↑	[145]
				Cleavage of caspase-3,-4 ↑, Bax ↑	
		22Rv1 cells	15 μg/mL, 24 h	PERK ↑, IRE1 ↑, CHOP ↑, GRP78/BiP ↑, Ero1 ↑, ER chaperone ↑, PDI ↑, XBP1 ↑, calnexin ↑	
				Cleavage of caspase-3,-4 ↑, Bax ↑	
*Asterella angusta*	Marchantin M	PC-3	10 μm, 48 h	GRP78/BiP ↑, CHOP ↑, XBP-1 ↑, p-eIF2α ↑,eIF2α ↑,ATF4 ↑, ATF6 ↑, ERAD ↓	[146]
				Cleavage of caspase-3,-4 ↑	
		DU145	10 μM, 48 h	GRP78/BiP ↑, CHOP ↑, XBP1 ↑, p-eIF2α ↑, eIF2α ↑, ATF4 ↑, ATF6 ↑, ERAD ↓	
				Cleavage of caspase-3,-4 ↑	
		LNCaP	10 μM, 48 h	GRP78/BiP, CHOP ↑, XBP1 ↑, p-eIF2α ↑, eIF2α ↑, ATF4 ↑, ATF6 ↑, ERAD ↓	
				Cleavage of caspase-3,-4 ↑	
*Monascus pilosus*	Monascuspiloin	PC-3	25 μM, 48 h	IRE1α ↑, p-eIF2α ↑, eIF2α ↑	[147]
	Quercetin	PC-3	150 μM, 48 h	Release of intracellular Ca^2+^ ↑, Δψm ↓, GRP78/BiP ↑, ATF4 ↑, IRE1α ↑ ATF6 ↑	[148]
				Bid ↓, Bcl-2 ↓, cleavage of caspase-12 ↓Bax ↑, PARP ↑, cytochrome c ↑, AIF ↑, Endo G ↑, cleavage of caspase-3,-8,-9 ↑	
*Zingiber zerumbet* Smith	Zerumbone	PC-3	30 μM, 24 h	Release of intracellular Ca^2+^ ↑, Δψm ↓, calpain ↑, GRP78, CHOP ↑	[149]
				cleavage of caspase-3,-7,-9 ↑, cleavage of PARP ↑, Bid ↑, Bcl-2 ↓	

ER associated degradation (ERAD).

**Table 6:** Bioactive compounds from natural products that induce ER stress-mediated apoptosis in liver cancer.

Family Name	Compound	Cell Line	Duration/Dosage	Mechanism	Reference
*Glycyrrhiza inflate*	Licochalcone A	HepG2	10 μM, 24 h	release of intracellular Ca^2+^ ↑, Δψm ↓, ATF6 ↑, eIF2α ↑, IRE1α ↑, CHOP ↑, GRP94 ↑, XBP1 ↑, GRP78/BiP ↑	[153]
				Cleavage of caspases-3,-4,-9 ↑, cleavage of PARP ↑	
				ROS ↑	
*Commiphora mukul*	Guggulsterone	Hep3B	30 μM, 12 h	Release of intracellular Ca^2+^ ↑, Δψm ↓, IRE1 ↑, JNK ↑, GRP78/BiP, PERK ↑, eIF2α ↑, ATF4 ↑, CHOP ↑	[154]
				DR5 ↑, cleavage of caspase-3 ↑, cleavage of PARP ↑	
				ROS ↑	
	Verrucarin A	Hep3B	1 μM, 12 h	GRP78/BiP ↑, p-PERK ↑, p-eIF2α ↑, CHOP ↑	[155]
				DR5 ↑, cleavage of caspase-3,-8 ↑, cleavage of PARP ↑	
				ROS ↑	
		HepG2	1 μM, 12 h	Chop ↑	
				DR 5 ↑	
	7-dimethoxyflavone	Hep3B	5 μmol, 24 h	CHOP ↑, GPR78/BiP ↑, ATF4 ↑	[156]
				DR5 ↑, cleavage of caspase-3,-8,-9 ↑	
				ROS ↑	
*Nelumbo nucifera* Gaertn	Neferine	Hep3B	20 μmol, 24 h	GRP78/BiP ↑, calnexin ↑, PDI ↑, calpain ↑	[157]
				cleavage of caspase-3,-6,-7,-8,-12, cleavage of PARP ↑, Puma ↑, BIM ↑, Bid ↑	
*Pycnostelma paniculatum* K Schum	Paeonol	HepG2	31.25 mg/mL, 24 h	GRP78 ↑, CHOP ↑	[158]
				cleavage of caspase-3 ↑, PI3K/AKT ↓	
*Salvia miltiorrhiza* Bunge	Cryptotanshinone	HepG2	10 μM, 24 h	eIF2α ↑, GRP94 ↑, GRP78/BiP ↑, cisplatin ↑, CHOP ↑	[117]
				cleavage of PARP ↑, JNK ↑, MAPK ↑	
				ROS ↑	
*Zingiber officinale* Rosc	6-Shogaol	SMMC-7721	20 μM, 6 h	GRP94 ↑, GRP78/BiP ↑, CHOP ↑, p-PERK ↑, PERK ↑, eIF2α ↑, p-eIF2α ↑	[159]
				cleavage of PARP ↑, cleavage of caspase-3 ↑	
		SMMC-7721 xenograft in Male SCID mice	6-shogaol (orally, 10 mg/kg), 28 days	p-PERK ↓, eIF2α ↓, p-eIF2α ↓	
				cleavage of caspase-3 ↑	
	Genistein	Hep3B	100 μM, 48 h	release of intracellular Ca^2+^ ↑, Δψm ↓, calpain ↑, CHOP ↑, GRP78/BiP ↑	[160]
				cleavage of caspase-2,-3,-7,-12 ↑, cleavage of PARP ↑, Apaf-1 ↑ cytochrome c ↑, Bad ↑	
				ROS ↑	
